# The contribution of qualitative research within the PRECISE study in sub-Saharan Africa

**DOI:** 10.1186/s12978-020-0875-6

**Published:** 2020-04-30

**Authors:** Marina A. S. Daniele, Melisa Martinez-Alvarez, Angela Koech Etyang, Marianne Vidler, Tatiana Salisbury, Prestige Tatenda Makanga, Peris Musitia, Meriel Flint-O’Kane, Tanya Wells Brown, Brahima Amara Diallo, Helena Boene, William Stones, Peter von Dadelszen, Laura A. Magee, Jane Sandall, the PRECISE Network, Umberto D’Alessandro, Anna Roca, Hawanatu Jah, Ofordile Oguchukwu, Andrew Prentice, Melisa Martinez-Alvarez, Brahima Diallo, Adbul Sesey, Kodou Lette, Alpha Bah, Chilel Sanyang, Marleen Temmerman, Angela Koech Etyang, Peris Musitia, Mary Amondi, David Chege, Patricia Okiro, Geoffrey Omuse, Sikolia Wanyonyi, Esperança Sevene, Paulo Chin, Corssino Tchavana, Salesio Macuacua, Anifa Vala, Helena Boene, Lazaro Quimice, Sonia Maculuve, Eusebio Macete, Inacio Mandomando, Carla Carillho, Peter von Dadelszen, Laura A. Magee, Meriel Flint-O’Kane, Rachel Craik, Amber Strang, Marina Daniele, Donna Russell, Tatenda Makanga, Liberty Makacha, Yolisa Dube, Newton Nyapwere, Lucilla Poston, Jane Sandall, Rachel Tribe, Andrew Shennan, Sophie Moore, Tatiana Salisbury, Ben Barratt, Lucy Chappell, Sean Beevers, Kate Bramham, Aris Papageorgiou, Alison Noble, Hannah Blencowe, Veronique Filippi, Joy Lawn, Matt Silver, Matthew Chico, Judith Cartwright, Guy Whitley, Sanjeev Krishna, Marianne Vidler, Jing Larry Li, Jeff Bone, Mai-Lei Maggie Woo Kinshella, Beth A. Payne, Domena Tu, Warancha Tumtaweetikul, William Stones

**Affiliations:** 1 Department of Women and Children’s Health, School of Life Course Science, Faculty of Life Sciences and Medicine, King’s College London, 5th Floor, Becket House, 1 Lambeth Palace Road, London, SE1 7EU UK; 2 Medical Research Council Unit in The Gambia, the London School of Hygiene & Tropical Medicine, London, UK; 3 Centre of Excellence in Women and Child Health, East Africa, Aga Khan University in East Africa, Nairobi, Kenya; 4 Department of Obstetrics and Gynecology, Faculty of Medicine, University of British Columbia, Vancouver, Canada; 5 Department of Health Service & Population Research, Institute of Psychiatry, Psychology & Neuroscience,, King’s College London, London, UK; 6 Department of Surveying and Geomatics, Faculty of Science and Technology, Midlands State University, Gweru, Zimbabwe; 7 MARCH Centre, London School of Hygiene and Tropical Medicine, London, UK; 8 Centro de Investigação em Saúde de Manhiça, Manhiça, Maputo Province Mozambique; 9 Department of Public Health and Department of Obstetrics & Gynaecology, Malawi College of Medicine, Blantyre, Malawi

## Abstract

The PRECISE Network is a cohort study established to investigate hypertension, fetal growth restriction and stillbirth (described as “placental disorders”) in Kenya, Mozambique and The Gambia. Several pregnancy or birth cohorts have been set up in low- and middle-income countries, focussed on maternal and child health. Qualitative research methods are sometimes used alongside quantitative data collection from these cohorts. Researchers affiliated with PRECISE are also planning to use qualitative methods, from the perspective of multiple subject areas. This paper provides an overview of the different ways in which qualitative research methods can contribute to achieving PRECISE’s objectives, and discusses the combination of qualitative methods with quantitative cohort studies more generally.

We present planned qualitative work in six subject areas (health systems, health geography, mental health, community engagement, the implementation of the TraCer tool, and respectful maternity care). Based on these plans, with reference to other cohort studies on maternal and child health, and in the context of the methodological literature on mixed methods approaches, we find that qualitative work may have several different functions in relation to cohort studies, including informing the quantitative data collection or interpretation. Researchers may also conduct qualitative work in pursuit of a complementary research agenda. The degree to which integration between qualitative and quantitative methods will be sought and achieved within PRECISE remains to be seen. Overall, we conclude that the synergies resulting from the combination of cohort studies with qualitative research are an asset to the field of maternal and child health.

## Background

### Introduction

The PRECISE Network (PREgnancy Care Integrating translational Science, Everywhere) has been established to investigate hypertension, fetal growth restriction and stillbirth (described as “placental disorders”) in Kenya, Mozambique and The Gambia, with core funding from UK Research and Innovation (UKRI). PRECISE’s major legacy will be the development of “highly-phenotyped” cohorts of women and the creation of a biorepository (von Dadelszen et al., The PRECISE protocol, in this issue). The study aims to learn about women in a 360° manner, by collecting data on their physical and mental health, nutrition, living environment and social conditions. These data will represent both individual-level co-exposures and contextual factors which may limit the local health services’ ability to effectively prevent, diagnose and manage these conditions. The aim is to identify the pathways that lead some women to develop placental disorders, and those which affect health outcomes for these women.

Cohort studies follow-up participants longitudinally and, thus, can be used to draw causal links between life-course exposures and health or disease. Pregnancy and birth cohort studies recruit participants (mothers, children, or both) antenatally or in early infancy, offering the potential to examine health outcomes in women of childbearing age and children. Some of these cohorts follow participants for many years, thereby enabling the study of how early-life experiences affect diseases of adulthood [1]. The majority use quantitative methodologies, however those that also include qualitative methodologies have the potential for additional insight into mechanisms between variables and why and how such mechanisms may be expressed along with additional understanding of the impact of context.

It is beyond the scope of this article to provide a comprehensive review of pregnancy and birth cohorts in LMICs, or to cite all those that have been accompanied by qualitative research. Instead, the aim of this section is to introduce major MCH-focused pregnancy and birth cohort studies that have combined quantitative and qualitative methods, using illustrative examples of a range of study designs from LMIC based on our prior knowledge of the field and on extensive literature searching.

Past and ongoing pregnancy and birth cohorts vary considerably in size, depending on the research question and power calculations, and may involve single or multiple countries. In LMICs, a limited number of large or multi-country pregnancy or birth cohorts have been conducted, due to the high costs and logistical challenges often involved [2, 3]. These include the Global Network Maternal Newborn Health Registry (280,000 pregnancies across 6 countries) [4, 5], the AMAHNI study (also 280,000 pregnancies, across 11 countries) [6] and the COHORTS consortium (11,000 children across 5 countries) [7], which have contributed high-quality data on MCH outcomes and coverage of key interventions, often from areas with poor health information systems. Other longitudinal multi-country studies have focussed on the development of international fetal and infant growth standards [8, 9].

While we could not find evidence of any qualitative research linked with the large multi-country cohorts cited above, with the exception of the Brazilian and South African contributors to COHORTS [10–15], several smaller or single-country studies have had at least one associated qualitative component [16–25]. Many of these can be considered mixed methods studies, defined as studies which combine both qualitative and quantitative approaches and involve a degree of mixing or integrating of the two, at one or more stages of the study (such as planning, data collection, analysis or interpretation). One of the two approaches may be dominant (often the quantitative), or they may have equal standing [26]. The core assumption behind a mixed methods approach is that the combination of the two approaches “provides a more complete understanding of a research problem than either approach alone” [27].

Some mixed methods cohort studies are conceived as such from the outset, with qualitative methods incorporated into the study protocol, and/or with qualitative and quantitative results reported in the same publication, suggesting a good level of integration [16, 17, 21, 23–25]. For example, a cohort study conducted in Burkina Faso with a nested ethnographic component investigated the health, social, and economic consequences of near-miss obstetric complications. The investigator team developed several publications, which variously presented certain quantitative findings alone [28], other quantitative findings together with some qualitative findings [16, 17], and other qualitative findings separately [29–31], suggesting integrated but independent lines of enquiry. In another example, 500 HIV-positive pregnant women in Uganda were followed longitudinally to assess status disclosure over time. The associated qualitative piece focussed on documenting experiences of, and barriers to, disclosure, and a joint publication was developed integrating the findings from the two components [25]. The latter is the case for yet another HIV-positive pregnancy cohort: in Kenya, 100 women were followed to study access to long-term anti-retroviral therapy, with a qualitative “sub-study” focussed on barriers and facilitators to navigating services [24].

There are also examples of cohorts which have planned and conducted qualitative research post hoc, following an identified need to further explore or explain emerging quantitative findings. This has been described as an explanatory sequential mixed methods design, in which the qualitative work is carried out after, prompted by the analysis of all or part of the quantitative data [27]. This was the case for the multi-country MAL-ED study on nutrition, infection and child health [22]. Following the observation that few mothers in the South African cohort were practicing exclusive breastfeeding by 3 months postpartum, the investigators initiated a qualitative study to explore mothers’ views on infant feeding [23].

Finally, some qualitative research is only loosely associated with a pregnancy or birth cohort. Therefore, can be considered to be linked, but separate, studies. The cohort principally serves as a sampling frame for the qualitative study, perhaps additionally contributing background information on the study participants. This may be the case when the cohort is followed for many years and the qualitative piece focusses on health issues that are relevant to participants as they get older. For example, two qualitative studies focussed on non-communicable diseases among older women were conducted two decades after the participants’ enrolment, together with their babies, in the Birth to 20 cohort study in South Africa [13–15].

## Main text

In this paper, we examine the different ways in which qualitative research methods can contribute to achieving PRECISE’s objectives. We present planned qualitative work within PRECISE in six subject areas and summarise the functions of this work in relation to the overall cohort study. Finally, we discuss the identified functions with reference to other MCH-focused cohort studies and in the context of the methodological literature on mixed methods approaches.

### Qualitative research within PRECISE by subject area

In this section we present the plans for qualitative research that have been developed by researchers affiliated with the PRECISE Network, with a focus on their relationship with the cohort study. While the PRECISE protocol refers to most of these topics at a high level, in most cases the research plans described below have developed organically and reached a higher level of definition during the first year of the project (von Dadelszen et al., The PRECISE protocol, in this issue). The work described is at different stages of completion at the time of writing (March 2019). For some subject areas, it is already underway, while for others implementation is conditional upon securing additional funding. The six subject areas that covered in this section are:

- Health systems
- Health geography
- Mental health
- Community engagement
- Qualitative assessment of the TraCer tool implementation
- Respectful maternity care

#### Health systems

Mothers and newborns need access to safe, efficient and high-quality health services along the maternal and neonatal health continuum of care, spanning the pregnancy, birth and the postnatal periods. This is particularly important for mothers and babies with placental disorders. To provide this care, health systems need to have efficient and equitable financing, adequately-trained and motivated human resources, effective governance and accountability structures, functioning referral pathways, sufficient physical resources and well-functioning information systems [32].

Within the health systems component of PRECISE, we plan to assess and compare the health system in the different country sites, with a focus on the ability to respond to obstetric emergencies and care for women with placental disorders and their babies. Based on Savigny and Adam’s 2009 framework for people centred health systems [33], we plan to:

- Examine the configuration of maternal and newborn health services, including which services are offered in which type of facility and by which type of personnel,
- Assess the availability and use of health services for mothers with placental disorders and their babies,
- Analyse human resources, financing and governance policies, including the different pathways through which health workers are trained to provide different types of care, the different sources of financing and the mechanisms through which funds are disbursed, and the upward and downward accountability policies in place to ensure quality care is provided, and
- Examine attitudes, practices, and beliefs surrounding pregnancy, childbirth and postnatal care in the local communities in relation to participation in the PRECISE cohort.

Our methods will include assessment of policy and guideline documents, semi-structured interviews with healthcare managers, health workers and postpartum women, as well as focus group discussions (FGDs) with women who are pregnant or have recently given birth, family members who would be involved in decision-making around pregnancy and birth, and community leaders, including members of community health boards and women’s associations. Data will be analysed using framework analysis [34] to create a map of the health system in each country, and through these comparisons draw recommendations on how health systems can best provide services for mothers and their babies. Qualitative methods will be complemented with a quantitative facility assessment, through which we will evaluate availability of drugs and medical supplies, number of human resources by cadre and level of training, and facility financing by source.

#### Health geography

Pregnancy outcomes may be influenced both by space (physical) and place (contextual) geographies. For example, physical distance to health facilities is a determinant of care seeking behaviours and mediates pregnancy-related outcomes [35, 36]. On the other hand, women may live in cultural contexts where they do not have the autonomy of financial decision-making concerning their pregnancy; this may increase their risk of adverse events.

We plan to use qualitative methods as part of the health geography component to gain an understanding of the local political and healthcare contexts (e.g. civil wars, natural disasters, foreign aid, micro finance etc.) affecting the communities under study, and how these could have had an impact on adverse pregnancy events [37]. In this process, as well as validating a predetermined set of socio-geographical indicators, we hope to identify new ones that elucidate the interactions between place and adverse outcomes. We will use qualitative methods in combination with other planned geospatial analyses involving maps and geographical information systems, to explore the spatial structure of the association between placental disorders and adverse outcomes [38].

To achieve these aims we plan to conduct FGDs with women of reproductive age, male decision makers, traditional healers, community health workers and health care professionals. Key community gatekeepers and leaders who give permission to proceed with the study will also be invited to participate in semi-structured interviews. Thematic content analysis will be used to analyse the data.

#### Mental health

Perinatal mental health disorders are a major public health challenge and contribute significantly to maternal mortality and morbidity [39]. Globally, it is estimated that only half of these disorders are identified. If left untreated, the negative consequences can have profound and lasting effects on women, children and the broader family. Limited research into perinatal mental health has been conducted across LMICs [40]. This has a significant impact on the ability of these countries to develop effective policies and services. The mental health work within PRECISE aims to provide insight into local concepts of perinatal mental health and identify the barriers and facilitators in the implementation of mental health screening in perinatal care in the three study countries.

We plan to conduct semi-structured interviews with pregnant and postpartum women to explore their perspectives and experiences of perinatal mental disorders. In addition, we will conduct FGDs with family members (e.g. partners, parents or parents-in-law), health providers and policy makers to understand local concepts of mental ill-health and its perinatal presentations. Qualitative work will be interpreted using a thematic network and step phased analytical approach [41]. With the permission of women, some stories will be drawn out and presented as individual case studies in relation to broader perspectives such as experiences of the health system, respectful care and life with a newborn. This will enable invaluable and deep understanding, appreciative of individual “cases” within the context [42]. The findings from this work will serve as a foundation for the adaptation, validation and integration of existing screening tools for use within PRECISE Network country sites. These adapted tools will enable the collection of quantitative mental health data, enabling investigation of associations between maternal mental health and placental disorders.

#### Community engagement

Community engagement is particularly important within PRECISE, given that the study populations vary widely in their traditions, socio-cultural beliefs and ways of life. In addition, they differ in their degree of exposure to previous research. Planned engagement activities include the consultation of community gatekeepers, the organization of public meetings, the publication of media broadcasts (jingles), and the consultation or setting up of community advisory boards. We plan to iteratively incorporate qualitative research methods into these activities to provide a formal structure for obtaining information and feedback from study participants and community representatives. In this context, qualitative methods will enable us to:

- Better understand local societies and cultures,
- Explore community perceptions on research and participation in research,
- Assess the (health) needs and expectations of the community, providing an opportunity to consider whether and how these could reasonably be met within the scope of the project,
- Determine the appropriate ways of approaching community gatekeepers (such as village elders, chiefs and religious leaders) and to establish suitably-composed advisory boards, and
- Determine the culturally appropriate processes for specific research activities such as using appropriate wording in communications with participants, gaining informed consent (e.g. the need to obtain household head permission in The Gambia) or handling biological samples such as the placenta and cord (Kenya).

The availability of these data will facilitate the establishment of a mutually respectful relationship between the research team and the study participants. In turn, this will be of concrete benefit to the study, for example by promoting compliance to follow-up within the cohort, or by facilitating the feedback of findings at later stages of the project. Comparative data analysis will focus on contextual similarities and differences between the sites. Analyses will inform the iterative improvements of the PRECISE initiative as well as the training program for data and bio-specimen collection. In addition, qualitative findings will aid the interpretation of other data e.g. survey data, for example by enabling us to identify (or discount) any “institutionalisation” of participants in sites with high levels of familiarity with the research team. Lessons learnt may also be useful to subsequent research studies in similar settings.

A variety of data collection methods will be employed: field notes and observations, FGDs, and in-depth interviews (IDIs). Some of these will be conducted alongside routine community engagement activities, while others will involve setting up separate sessions specifically for qualitative data collection, to address specific research questions. Themes will be developed by inductive content analysis of the data in an iterative process.

#### Qualitative assessment of the TraCer tool implementation

Reliable estimation of gestational age (GA) is an important component of any pregnancy cohort. GA assessment improves the quality of the research data but is also valuable clinically in informing decision-making in maternal illness and neonatal care. PRECISE is keen to employ GA assessment methods that are suited to the contexts where the research is taking place. To this end, we are developing a prototype mobile health application – the TraCer tool – that automatically ‘captures’ and measures the transcerebellar diameter (TCD) to obtain the GA. The TCD can be used to estimate GA from the early second trimester and is resilient to most forms of fetal growth restriction. Women in many parts of sub-Saharan Africa present for antenatal care late in pregnancy, when standard methods of GA assessment are associated with considerable inaccuracy. We hope that the tool will be suitable for use by relatively low skilled healthcare workers with little training, and that this would be easier to scale up and sustain in LMICs compared to full complete ultrasound assessment.

We plan to employ qualitative methods including FGDs and IDSs both before and during the introduction of the tool, to seek to understand the value of GA assessment to the women, their families and communities in the research settings and to the health care workers and managers as well. The team will seek to evaluate the acceptability and feasibility of using TraCer beyond the research setting for routine GA assessment in antenatal care. In addition, we plan to use structured observations to collect data on the usability of the tool. Analyses will inform iterative improvements of the TraCer tool and development of the training program and manual for its use in the field.

#### Respectful maternity care

Most women now give birth in health facilities in many LMICs [43], and their experience of maternity care has been the focus of increasing attention over the past decade. The provision of respectful maternity care (RMC), and the absence of mistreatment in facilities (also known as disrespect and abuse or D&A), have been framed both as quality of care issues, and as a human rights concern [44–46]. Despite recently receiving a high level of academic interest, there are still important research gaps in this field. As a multi-country longitudinal study, PRECISE provides us with a timely opportunity to address these. Within the RMC stream, we plan to use qualitative methods for two main purposes.

The first is to gain a nuanced understanding of how women who suffer pregnancy complications and adverse perinatal events (maternal near-miss, stillbirth, perinatal death, newborn illness or prematurity) experience care for themselves and their babies in the PRECISE sites, and how providers experience caring for these women, using semi-structured interviews. Another knowledge gap concerns what constitutes RMC/D&A of the newborn [47]. Therefore, we plan to focus a part of the interviews on how women perceive that their baby (whether alive or stillborn) was treated, and on the corresponding experience of health workers. Finally, as there is a need for more evidence on the drivers of RMC/D&A and for ideas for interventions [48], we plan to ask participants what conditions, in their experience, facilitate the provision of RMC. We will consider the need to use additional methods including FGDs and observations of care.

The second purpose of planned qualitative work is to support the adaptation and validation of an existing survey instrument with the aim of capturing RMC/D&A of the newborn. The long-term aim would be to administer this survey to the PRECISE cohorts, in order explore associations between the experience of care and health outcomes. We plan to use qualitative interviews to test face-to-face content validity with postpartum women, as well as with international experts in maternal and newborn health.

### The functions of qualitative work within PRECISE – a summary

Based on the plans outlined above for each subject area, we have extrapolated a list of the different functions of qualitative work within the PRECISE network. Within each subject area, qualitative work may serve one or more functions. These are spelled out in Table 1 and will be discussed below.

**Table 1:** Functions of qualitative research within PRECISE, by subject area

Functions of qualitative research within precise	Health systems	Health geography	Mental health	Community engagement	TraCer	Respectful care
a. Provision of contextual information to support quantitative cohort implementation				✓		
b. Provision of contextual information to be integrated in the interpretation of overall cohort findings	✓	✓		✓		
c. Assessment of the acceptability of technology to be used in quantitative cohort					✓	
d. Validation/adaptation of indicators/measures for quantitative cohort data analysis		✓	✓			
e. Independent qualitative or mixed methods agenda - defined as intention to publish at least some qualitative results separately from the main quantitative cohort findings	✓	✓	✓	✓	✓	✓
f. Identification of ideas for intervention development	✓					✓
g. Validation/adaptation of indicators/measures for independent mixed methods research agenda						✓

## Discussion

In this section, we discuss the identified functions of qualitative research in PRECISE with reference to similar cohort studies, where possible using LMIC examples, and in the context of the methodological literature on mixed methods research.

One of the main purposes of combining qualitative and quantitative methods is the use of the results from one method to help develop or inform the other, for example in the areas of implementation and measurement decisions [49]. This qualitative research is usually conducted before the quantitative work takes place, and informs it [27]. Functions a., c. and d. in PRECISE speak to this purpose. Firstly, research in the area of community engagement will inform the practical aspects of setting up the cohort (function a.). Similar uses of qualitative work can be seen in other cohorts. Quoting two non-LMIC but eloquent examples, researchers interviewed women about their decision to participate (or not) in pregnancy or birth cohorts in the UK and Canada, with the aim of addressing barriers to recruitment in future studies [50, 51]. Secondly, in terms of measurement decisions, functions c. and d. represent the ways in which qualitative methods will contribute to the validation of indicators, measures, or technology. Although these pieces are intended to inform data collection in PRECISE, their results are likely to be beneficial beyond this study. Similarly, the validation of the Edinburgh Postnatal Depression scale in Brazil was done to inform data collection in the Pelotas birth cohort, but will certainly be useful for other research [12].

Function b. in PRECISE involves the collection of qualitative data concurrently with the cohort study, with the expectation that the findings will be integrated in the interpretation of the overall results, including the quantitative findings. This is a common purpose of qualitative research conducted alongside pregnancy and birth cohorts. For example, cohort studies focussed on pregnant women’s use of gestational diabetes screening and treatment in India [21], or of HIV services in Kenya [24], have used qualitative methods to provide a more in-depth understanding of barriers and facilitators that may affect access to those services. In Benin and Burkina Faso, qualitative research has provided a deeper understanding of the effect of obstetric complication and near-miss, the focus of the cohort studies, on women’s lives [16, 18, 52]. In PRECISE and in these examples, the qualitative and quantitative components aim to answer related research questions, in pursuit of a more comprehensive, holistic and contextual understanding of a single phenomenon. This is based on the assumption that combining different methods allows researchers to exploit the assets of each one neutralise, rather than the compound, their liabilities [53].

Combining method-specific findings at the interpretation stage is often done with the aim to seek convergence, corroboration, or essentially cross-validation between these findings. This is known as triangulation [54]. When instead the purpose is for one method (usually the qualitative) to elaborate, enhance, illustrate or clarify the results of the other (usually the quantitative), this is known as complementarity [49]. One concern is that researchers within each PRECISE subject area may have not yet anticipated whether they expect their qualitative findings to be used in triangulation or complementarity with the quantitative findings. It is important to consider that combining qualitative and quantitative approaches may lead to incommensurable or contrasting findings, especially if the methods we have chosen stem from different paradigms and methodologies [55]. Should this occur, the risk is that the quantitative findings will dominate [56, 57].

At the heart of these considerations is the fundamental question as to whether PRECISE should be considered a mixed-methods project, which is related to the expected degree of integration between the methods throughout the study. We are raising this issue here, but do not propose a definitive answer. While PRECISE is planning to deliver a “broad programme of holistic, interdisciplinary pregnancy research” (von Dadelszen et al., The PRECISE protocol, in this issue), the multiplicity of the workstreams and plurality of approaches within a single research project mean that not every aspect of method integration has yet been established and planned. Based on their current plans for qualitative work, there appear to be differences between the subject areas with regard to whether and at which point (epistemological, data collection, analysis or write up) integration with the quantitative cohort study has already occurred or is desired and achievable in the future. Difficulties in achieving integration, and the resulting need to resort to reporting on each component individually, are common problems in mixed-methods studies [58].

PRECISE may perhaps be best described as a platform upon which other, related studies can “piggy-back”. Within all the subject areas covered in this paper, researchers are planning to publish at least some of their qualitative findings separately from the quantitative publications related to the cohort, suggesting that co-existence within the Network of independent qualitative research agendas (function e.). Within the health systems and respectful care areas, there is an additional aspiration to use the findings to inform the development of interventions (function f.). This is also a feature of other pregnancy or birth cohorts. For example, a USA qualitative study exploring cultural understandings of pelvic floor support, conducted alongside a cohort study of postpartum women, will inform the development of a toolkit to support postpartum recovery [59]. Similarly, a UK study on mental health among fathers of birth cohort participants aimed to inform options for service provision for this group [60].

The lack of core funding for some of these of the pieces described means that in several cases these plans must be considered aspirational rather than concrete. In general, the breadth of PRECISE as a platform poses practical challenges in terms of setting research priorities and allocating funds. However, the potential breadth of data that can be collected and variously compared and combined is impressive. Importantly, if, as we hope, all these plans are implemented, they will bear witness to PRECISE’s achievement of its objective to develop individual and institutional capacity in research strategy and delivery across the Network. In addition, the ambition to include multiple research methods within one study is commendable as it can bring notable scientific advantages. Mixing methods encourages researchers to think outside the box of their specific discipline and be open to the input of others. It stimulates them to move beyond their usual, comfortable position on one or other side of the macro/micro or quantitative/qualitative divide, and to frame research questions “whose aim is precisely to focus on how different dimensions and scales of social existence intersect or relate” [57]. Fruitful synergies between qualitative and quantitative methods will be possible on condition that we, as researchers, succeed in embracing their complexity and avoid underplaying the differences between approaches in pursuit of neatly consistent results.

## Conclusion

In conclusion, we have presented plans for qualitative work in six subject areas within PRECISE, and discussed these with reference to other MCH-focussed cohort studies and in the context of the methodological literature on mixed methods approaches. Qualitative research may have several different functions in relation to the cohort with which it is associated. It may directly inform the quantitative cohort data collection or interpretation, or be more focused on fulfilling an independent research agenda. The degree to which integration between qualitative and quantitative methods will be sought and achieved within PRECISE remains to be seen. Overall, we conclude that the synergies resulting from the combination of cohort studies with qualitative research are an asset to the field of maternal and child health.

## Abbreviations

D&A: Disrespect and abuse
FGD: Focus group discussion
GA: Gestational age
IDI: In-depth interview
LMIC: Low- and middle-income country
MCH: Maternal and child health
PRECISE: PREgnancy Care Integrating translational Science, Everywhere
RMC: Respectful maternity care
TCD: Trans-cerebellar diameter
TraCer: The trans-cerebellar tool
UKRI: UK Research and Innovation
